# Tolerogenic Dendritic Cells and T-Regulatory Cells at the Clinical Trials Crossroad for the Treatment of Autoimmune Disease; Emphasis on Type 1 Diabetes Therapy

**DOI:** 10.3389/fimmu.2019.00148

**Published:** 2019-02-06

**Authors:** Brett Eugene Phillips, Yesica Garciafigueroa, Carl Engman, Massimo Trucco, Nick Giannoukakis

**Affiliations:** ^1^Allegheny Health Network Institute of Cellular Therapeutics, Allegheny General Hospital, Pittsburgh, PA, United States; ^2^Department of Biological Sciences, Carnegie Mellon University, Pittsburgh, PA, United States

**Keywords:** tolerogenic dendritic cells, T-regulatory cells, type 1 diabetes, clinical trials, immunotherapy, autoimmunity

## Abstract

Tolerogenic dendritic cells and T-regulatory cells are two immune cell populations with the potential to prevent the onset of clinical stage type 1 diabetes, and manage the beginning of underlying autoimmunity, at the time-at-onset and onwards. Initial phase I trials demonstrated that the administration of a number of these cell populations, generated *ex vivo* from peripheral blood leukocytes, was safe. Outcomes of some of these trials also suggested some level of autoimmunity regulation, by the increase in the numbers of regulatory cells at different points in a network of immune regulation *in vivo*. As these cell populations come to the cusp of pivotal phase II efficacy trials, a number of questions still need to be addressed. At least one mechanism of action needs to be verified as operational, and through this mechanism biomarkers predictive of the underlying autoimmunity need to be identified. Efficacy in the regulation of the underlying autoimmunity also need to be monitored. At the same time, the absence of a common phenotype core among the different dendritic cell and T-regulatory cell populations, that have completed phase I and early phase II trials, necessitates a better understanding of what makes these cells tolerogenic, especially if a uniform phenotypic core cannot be identified. Finally, the inter-relationship of tolerogenic dendritic cells and T-regulatory cells for survival, induction, and maintenance of a tolerogenic state that manages the underlying diabetes autoimmunity, raises the possibility to co-administer, or even to serially-administer tolerogenic dendritic cells together with T-regulatory cells as a cellular co-therapy, enabling the best possible outcome. This is currently a knowledge gap that this review aims to address.

## Introduction

Type I diabetes (T1D) is a progressive autoimmune disease resulting in the impairment and loss of pancreatic insulin-producing beta cells via innate and adaptive leukocyte activity (1). The resulting dysregulation of, and eventual loss of controlled blood glucose variability, facilitates the onset of disease-associated complications like cardiovascular, neurologic, ophthalmic, and renal complications. T1D is a managed disease in need of a cure and despite the investment made in novel insulin formulations and glycemia level-activated pumps, pharmacologic insulin replacement fails to achieve a return to stable and long-term physiologic glycemic variability, to avoid the onset of the complications (2–7). Stem cell-based insulin-producing surrogate cells for transplantation are still far from being a realistic clinical option, also presenting their own challenges (8, 9). Similarly, islet allo- or xeno-transplantation, in spite of its clinical success, is applicable only for a select and very-restricted patient category (10, 11) with its own limitations conferred by an allogeneic or xenogeneic immune response on top of a latent autoimmunity that is readily re-activated.

## The Points of Action of Tolerogenic Dendritic Cells

**Figure 1:**
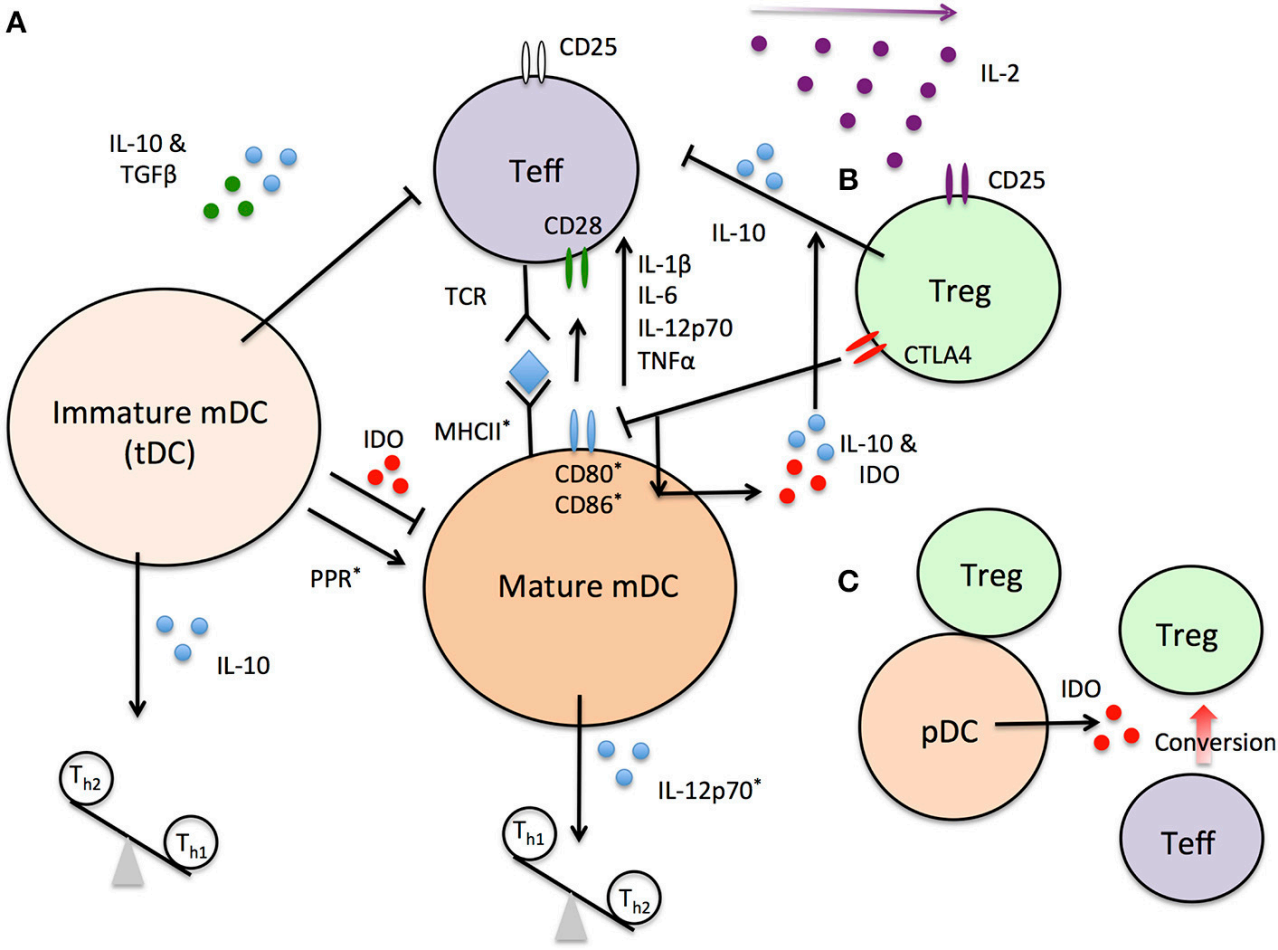
A simplified schematic of DC and Treg interactions. **(A)** immature mDC secrete anti-inflammatory cytokines inhibiting Teff activation and driving Th_2_ differentiation. Pattern recognition receptor (PPR)-dependent maturation of mDC increase expression of ^*^-labeled molecules required for Teff primary and secondary activation. Changes in cytokine expression profiles further drive Teff activation and tip the T_h_ balance toward Th_1_ cells. **(B)** treg can block Teff activation directly or through indirect interactions with mature DC. Treg also preferential sequester the T-cell proliferation factor IL-2 due to high constitutive IL-2R (CD25) expression. **(C)** pDC/Treg interactions stabilize and convert Teff to Treg populations in lymph nodes under steady state conditions.

Dendritic cells (DC), alone or via T-regulatory cells (Tregs) and B-regulatory cells (Bregs), can determine the state of activation and can even direct the differentiation of pro-inflammatory and autoreactive CD8+ cytotoxic T-cells (CTL) as well as the balance of T-helper cell (TH)1, TH2, and TH17 populations (12–19) (Figure 1). Even though the different tolerogenic dendritic cell (tDC) populations used in clinical trials for autoimmunity thus far, including T1D, are mainly of the myeloid lineage (12–15), it has far from conclusively-demonstrated that they represent a completely-pure myeloid-derived lineage, following *ex vivo* generation, in the widely-used granulocyte-macrophage colony-stimulating factor (GM-CSF) + interleukin-4 (IL-4) cell culture medium (20). There is an important gap-in-knowledge concerning the actual balance of plasmacytoid DC (pDC), type 1 myeloid DC (mDC), and type 2 mDC (21–28) as well as what can be naturally-tolerogenic DC populations (20) inside the *ex vivo*-generated cell products immediately following generation under GMP conditions and even more so immediately prior to the time of administration. It is also unclear if such a potential balance changes immediately following administration at the site of injection (usually intradermal/sub-cutaneous injection), or after DC migration into the lymphoid organs draining the site of injection. There are intriguing data indicating that *ex vivo*-generated tolerogenic DC (tDC) seeding and remaining inside the administration site are associated with the development of a neo-lymphoid stroma inside which Tregs, expressing the Foxp3 transcription factor (Foxp3+ Tregs), emerge (29). The significance of this event for the overall tolerogenic outcome, post-tDC treatment in autoimmunity, and in T1D in particular, remains to be determined. Studies in mice have largely focused on the expression at the cell surface of the common co-stimulation proteins on exogenously-administered tDC recovered from the lymph nodes draining the administration site, as well as the immunokines they produce, yet none of these phenotypes/activities have yet been associated with actual tolerogenic activities, resulting in direct or indirect suppression of autoreactive T-cells transiting through these organs. This, we believe is an important and unaddressed area of research which should be pursued to more-completely understand how tDC can affect the activation of effector T-cells inside the lymph nodes. It is possible that a common outcome on such effector T-cell activity could be identified and subsequently be associated with a measurable biomarker in peripheral blood or other easily accessible biofluid. Although a reasonable amount of data suggests tDC are able to maintain their tolerogenic state in the face of pro-inflammatory signals (30–33), there are some data that suggests that this is not always the case (34, 35). Whether low co-stimulation potential *in vivo* is *conditio sine qua non* for tDC, to confer some form of regulation and activity arrest in effector autoreactive T-cells inside the lymphoid organs, therefore remains an open question in terms of if it is critical in the mechanism of action of tolerogenic DC.

## Are There Points of Intersection in Phenotype and Tolerogenic Activity Among the Different Clinical Tdc Products Tested in Trials To-Date?

In order to address this question, the different methods currently-used to generate tDC *ex vivo*, must be considered. Immature DC are generated from isolated monocytes with the addition of IL-4 and GM-CSF to the culture media, a method that is shared between clinical therapeutic techniques. To enforce or impart additional tolerogenic properties to the DC, various other agents have been used that impair DC maturation or specific pro-inflammatory functions (vitamin D3, immunosuppressive Dexamethasone and NF-κB inhibitors, antisense oligonucleotides targeting co-stimulatory molecules) (20) (Table 1). tDC have been utilized to reduce tissue transplant rejection (36–39) and treat autoimmune disease (20, 40), the latter of which has utilized disease specific auto-antigens to enhance immune tolerance functions of tDC. To what extent these conditions change cellular effectiveness and mechanisms of action of tDC to confer their potentially beneficial effects, is unclear at present.

**Table 1:** A comparison of current protocols for *ex vivo* generated tDC and Treg and their clinical application.

**Cell source**	**PBMC**	**PBMC**	**PBMC**	**PBMC**	**PBMC**	**Umbilical cord blood**	**PBMC**
Target cell	DC	DC	DC	DC	Treg	Treg	Treg/Tr1
Cell generation	GM-CSF+ IL-4 & Anti-sense CD40, CD80, CD86	GM-CSF+ IL-4 & BAY 11-7082 Auto-antigens	GM-CSF+ IL-4 & Dex Vitamin D3 MPLA	GM-CSF+ IL-4 & Dex Vitamin A Cytokines	IL-2 Anti-CD3 & CD28 Beads	IL-2 Anti-CD3 & CD28 Beads	IL-2 IL-4 Anti-CD3 antibody Ovalbumin
Added auto-antigens	No	Yes	No	Yes	No	No	No
*Ex vivo* Cell characterization	Low CD40 CD80 CD86 IL-12	Low CD40 CD80	Low CD83 IL-12 High CD86 IL-10	Low CD83 IL-12 High CD80 CD86 IL-10	Low CD127 High CD25 Foxp3	Low CD127 IL-2 IFNγ High CD25 Foxp3 CD39	Low CD62L CD127 IL-4 IFNγ High Foxp3 CD25+ IL-10 IL-13
*In vivo* application	Increased Foxp3 Tregs IL-10 Bregs IL-4 IL-10 No Change DC	Increased Foxp3 Tregs Decreased IL-15 IL-29	No Change Foxp3 Treg	Increased Foxp3 Tregs	Increased Foxp3 Tregs Not Examined DC	X	X

*A brief listing of reported cellular characteristics of generated cells are shown, but have not been uniformly examined across all studies. Post-administration changes in cell populations and plasma cytokines in vivo, in patients, are listed. Increased Treg numbers are reported in a majority of trials that utilize either ex vivo generated autologous tDC or Treg. Techniques marked as “X” are in clinical trials but have only been published under conditions with pre-clinical settings*.

A major coordinator of pro-inflammatory gene expression and DC maturation is the transcription factor NF-κB. DC grown in the presence of NF-κB-inhibiting compounds, displayed a reduced expression of CD40 and HLA-DR (human leukocyte antigen-antigen D related, a class II HLA molecule) (41–43). Generation of tDC under GM-CSF+IL-4 conditions, result in suppressed NF-κB transcriptional activity (32, 43–47), which may be one avenue whose outcomes could identify a common tDC phenotype and the state of activity that informs mechanisms of the action of effector autoreactive T-cells. In the Rheumavax clinical trials (13) for rheumatoid arthritis, tDC generated in GM-CSF+IL-4 and the NF-κB inhibitor Bay 11-7082, exhibited lower CD40, and HLA-DR on a per cell basis (13). In the AutoDECRA trials (12, 32), dexamethasone (Dex) and vitamin D3-gerated tDC, were characterized with a low surface expression of the co-stimulation proteins CD40 and CD86 and the DC maturation marker CD83, with low levels of cell surface HLA-DR and very low concentrations of secreted IL-12p70 (32, 43, 45, 46). Instead, these tDC produced high concentrations of the immunosuppressive IL-10 immunokine (48). Interestingly, Vitamin D3 in addition to DC *in vitro*, can also achieve similar outcomes (47, 49, 50). Another approach to generate tDC relied on the addition of Dex, vitamin A, IL-1β, IL-6, tumor necrosis factor alpha (TNFα), and prostaglandin E2 in the culture medium (15, 51). The cell products exhibited elevated CD80 and CD86, and low CD83 expression. The MERTK gene product, a glucocorticoid-induced receptor that is prevalent in tDC, was also expressed at high levels. Production of IL-10 was detected in the cells with no detectable IL-12p70 or IL-23 in the cell culture media. Allogenic mixed lymphocyte reactions, performed in the presence of tDC, resulted in low T-cell proliferation and interferon gamma (IFNγ) production.

In our approach to treat T1D, we have generated tDC using a targeted approach; to directly impair the expression of three key co-stimulation proteins at the cell surface by *ex vivo* exposure of GM-CSF+IL-4-generated DC, to a mixture of antisense phosphorothioate DNA oligonucleotides, targeting the 5′ end of the primary transcripts of CD40, CD80, and CD86 (14). Removal of these co-stimulatory molecules resulted in incomplete T-cell activation during DC antigen presentation inducing anergy (52–54). In addition to a significant reduction in cell surface levels of the proteins *ex vivo*, these tDC also exhibited some other interesting features, previously reported (55), that involve potential aptameric effects through secondary and tertiary nucleic acid structures on toll-like receptor 9 (TLR-9) signaling on the activity of phosphatidylinositol-3-kinase and glycogen synthase kinase-3β, that are still under mechanistic investigation.

## Exogenously-Supplied Autoantigens or Autoantigen-Derived Peptides: Are They Necessary?

Autoimmune diseases each have their own unique auto-antigens and associated self-reactive T-cell populations. Preloading tDC with specific disease antigens, in some, but not all instances, enhance their ability to directly interact and inactivate self-reactive T-cells that cause tissue damage (56–58). Methodically this technique should reduce the chance of inducing tolerance to non-specific antigens and may provide a stronger suppressive effect of tDC for disease treatment. In instances where autoantigens are well-defined, peptides could be used in their native or post-translationally-modified, autoantigenic form. For example, tDC for multiple sclerosis treatment considerations, have been generated in the presence of GM-CSF+IL-4, Dex (or Vitamin D3) and pre-loaded with myelin self-peptides (20). In one of the Rheumavax studies, GM-CSF+IL-4 culture medium was supplemented with the NF-κB inhibitor Bay 11-7082 and then the cells were exposed to citrullinated peptides of aggrecan, vimentin, collagen type II and Aα and Bβ fibrinogen, which are putative RA autoantigens (59). The rationale for this method and approach to generate rheumatoid arthritis-specific tDC, was based on the findings that anti-citrullinated protein antibodies are found in 50–80% of patients over the lifetime of the disease (60). Not all patients, however, display uniform self-antigens for a given disease. In T1D, for example, a range of self-antigens and auto-antibodies are differentially-produced among patients and at different points during disease progression (61–64). Although most experts in the field of T1D autoimmunity pathogenesis agree that insulin and GAD65 are the major T1D auto-antigens, and therefore, by adding peptides from these proteins at the time of tDC generation could provide some level of antigen-specificity in terms of what populations of autoreactive T-cells are suppressed, the same experts note that by the time of disease onset, a significant degree of antigen spreading has occurred where other “late-antigen”-specific T-cells may in fact be driving autoimmunity. Targeting only common antigens may lead to reduced or abrogated effectiveness of tDC treatments, as has been demonstrated in at least one T1D animal model (57). Screening patient's autoantigen and self-reactive T-cell profiles are possible, although expensive, and still does not guarantee that each individual's responsible antigen is known, as the list of diabetes antigens continuously grows. The possibility of using individualized autoantigen profiles was addressed in the Newcastle study which used tDC for the treatment of rheumatoid arthritis. Synovial fluid contents from inflamed joints of each patient were added to the generated tDC, followed by *in situ* administration of the tDC into the inflamed space where, presumably, the cells would acquire patient-specific auto-antigens (12, 32). At this time though, the use of autoantigen loading in diabetic tDC treatments seems premature, with the ongoing discovery of new autoantigens and the lack of a concentrated biofluid that could serve as a natural reservoir of patient specific autoantigens.

## Non-cellular Factors as tDC-related Disease Modifiers

Two major differences among the clinical trials using tDC, lie in the manner in which they are administered. This could affect what kinds of mechanisms are activated to suppress autoreactive T-cells and to slow down, if not altogether halt disease progression. The first difference lies in the dose level administered. The second difference lies in the selection of the site of administration. This difference is important, we believe, in the kind of mechanism tDC activates, especially as the sites of inflammation and the cell populations constituting the inflammatory cells are different among autoimmune diseases. The majority of tDC clinical trials to date, consider local cell administration at the site which is subserved by lymph nodes that co-incidentally drain the site of inflammation, with the objective of facilitating tDC migration into the draining lymph node. Lymph nodes that drain the site of inflammation of an organ- or tissue-restricted autoimmune disease are characterized by a notable frequency of activated self-reactive T-cells, that are potential targets for anergy induction (65). Examples include the administration of tDC to an area subserved by the cervical lymph nodes in a recent multiple sclerosis trial (clinicaltrials.gov identifier: NCT02618902) and abdominal administration of tDC proximal to the pancreas in our T1D trial (14). An alternative approach is to directly introduce tDC into the site of inflammation proper, bypassing any consideration of lymphoid organ drainage. An example that has been suggested is the direct administration of tDC to actual inflamed sites in Crohn's disease (15). While the Newcastle University rheumatoid arthritis study introduced tDC directly at the site of inflammation, the intended goal was still for the migration of tDC to local draining lymph nodes. Even though the technique is more invasive than intradermal administration to facilitate tDC trafficking to the lymph nodes co-draining the inflamed tissue and the site of administration, the introduction of tDC producing IL-10 may have the added benefit of local immunosuppression inside the site of inflammation. This consideration is balanced by the possibility of an unwanted adverse effect where local inflammatory conditions may alter the phenotype of the *ex vivo* administered tDC, toward a more pro-inflammatory state.

## What, Then, are the Common Phenotypes and Activities?

In Table 2, we provide a list of markers that reliably distinguish the cells listed in the first column and that could be helpful to distinguish clinically-useful tDC from non-regulatory DC population during and after the cell generation process *ex vivo*. Of the tDC populations generated under different conditions, only four have entered clinical trials in autoimmune disease with outcomes publicly-reported (12–15). NF-κB inhibition is the central feature of at least three of these tDC populations. Other features shared in common by these different tDC populations include decreased CD83 expression, decreased IL-12 secretion, and elevated IL-10 secretion. Even though a common phenotype, other than suppressed NF-κB activity and perhaps low concentrations of pro-inflammatory immunokines, cannot be used as a distinguishing cell-inherent feature of tDC, all tDC share one mechanistic feature: increased regulatory lymphocytes (e.g., Foxp3+ Tregs and Bregs) *in vivo*, in animal models of autoimmune disease as well as in the peripheral blood of patients following administration (13–15). In addition to increased numbers of Foxp3+ Tregs in the circulation and inside the lymph nodes draining the injection site, there are reports of increased Bregs as well (17, 66). We noted that increases in patient C-peptide levels are correlated with B220+ CD19+ CD5+ CD1d+ IL-10+ B Bregs in the patients treated with our tDC (14).

**Table 2:** Cell marker and cytokine profiles for tolerogenic cell populations and mature dendritic cells.

**Cell type**	**Makers**	**Cytokines**	**References**
Immature mDC GM-CSF & IL-4	CD1c+ CD11c+ CD14- HLA-DR^Low^ CD40^Low^ CD80^Low^ CD83^low^ CD86^Low^	IL-10, TGFβ, IL12p70-	(81, 83–85)
Mature mDC	CD1c+ CD11c+ CD14- HLA-DR^High^ CD40+ CD80^High^ CD83^High^ CD86^High^	IL-12p70^High^	(86–88)
DC-10	CD1c- CD14+ CD16+ CD11c+ HLA-DR+ CD83+ CD68- CCR7+	IL-10^High^, IL-12p70-	(89, 90)
Treg	CD3+ CD4+ CD25+ CD127- Foxp3+ CTLA4+	IL-10^Low^	(89, 90)
Tr1	CD4+ CD49b+ LAG-3+ CD226+	IL-10^High^, TGFβ	(70, 90)

*In Treg cells, the lack of CD127 is used as a surrogate extracellular marker for the intracellular Foxp3 with 98% accuracy (68)*.

Much of the current divergence among different tDC populations, in terms of phenotype and points of mechanistic intersection, other than their ability to confer an increased frequency of regulatory immune cells in the peripheral blood and/or the lymph nodes draining their site of administration, might also be due to the *ex vivo* upstream cell processing procedures prior to the addition of GM-CSF/IL-4. Examples include the degree of “contaminating” monocyte progenitors and granulocytes in the monocyte elutriation step(s). The effect of the site of delivery (intravenous, subcutaneous, intradermal) on tDC mechanism of action (direct or indirect), at the lymphoid organs draining the inflamed tissues and/or the autoimmunity target tissues proper, remains to be better understood. In this light, establishment and retention of a tolerogenic phenotype can be a function of the *ex vivo* generation procedure and the method of/site of administration. Even once standardized methods are established to characterize an autologous *ex vivo*-generated cell population as tolerogenic, together with a set of biomarkers to confer such a designation, the ability of such cells to maintain proper function before and after administration will need to be verified and validated in human trials. Cellular therapies may require multiple injections over an extended period of time in some or all individuals. Generating and testing a single large batch of cells per patient could prove more cost effective than having several rounds of peripheral blood collection and differentiation, but storage methods, shelf-life, and frequency of retesting need to be determined. It is important, at the same time, to determine if freshly generated vs. thawed cryopreserved tDC are functionally-different *in vivo*. The objectives of international collaborations like the ones resulting in the proposal of tDC and Treg MITAP (Minimum Information about Tolerogenic Antigen-Presenting cells) are commendable steps in establishing uniform characterization of clinical tolerogenic cell products (67).

## Tregs as a Common Mechanistic Outcome of tDC Administration and as a Basis of Management of the Underlying Autoimmunity in Autoimmune Disease, Including T1D

In autoimmunity, Tregs induce tolerance through indirection consequences of physical interaction with DC, or through direct regulation of autoreactive T-helper and/or T-effector cells. While representing a population of cells that are diverse in character and phenotype, Tregs largely refer to cells that are mainly CD4+ CD25+ CD127- Foxp3+ (68, 69) as well as CD4+ CD49b+ LAG-3+ CD226+ IL-10 producing cells (Tr1 cells) (19, 70, 71). Treg constitutively express the surface marker Cytotoxic T-Lymphocyte-Associated protein 4 (CTLA-4), which is able to interact with DC co-stimulatory molecules CD80 and CD86. This not only acts as a competitive inhibitor blocking T-effector cell activation through CD28, but in a reciprocal manner on DC, which causes their expression of IDO, TGFβ, and IL-10, further amplifying the tolerogenic state of DC and the suppressive activity of the Tregs (72, 73). IL-10 and TGFβ are also produced from Treg cells blocking T-effector activation, with greater levels of production in Tr1 cells than Foxp3+ Treg cells. Mechanistically, Treg also compete with T-effectors for the cytokine IL-2, a necessary growth factor for cell proliferation and maintenance. Tregs constitutively express high levels of the IL-2 receptor α chain (CD25), which is the ligand-binding part of the IL-2 receptor complex. Thus, at limiting concentrations of IL-2, Tregs will sequester a greater amount of IL-2 away from T-effector cells.

## *Ex vivo*-Generated Tregs for the Treatment of T1D Autoimmunity

Given the strong evidence demonstrating powerful suppressive activities of stably-expressing Foxp3+ Tregs on autoimmunity, their consideration for clinical translation was self-evident early on. The first major hurdle in cell-based therapeutics is coming to a consensus on what is known and what is yet to be clarified, in order to move forward in therapeutic development. Foxp3+ Tregs are better characterized than tDC, with a defined marker profile of CD3+ CD4+ CD25+ CD127^low^. Furthermore, changes in CD25 expression levels and increased STAT5 pathway activity prior to administration to patients have been identified in clinical studies. Although their absolute numbers are low in the peripheral blood of humans, a number of techniques have evolved for their *ex vivo* expansion (74–77). Besides differences in the concentration of IL-2 supplied to the *ex vivo* Treg generation culture media, the current methods to expand Treg are consistently uniform and somewhat reproducible for future trials.

The greatest challenge and point of uncertainty is what happens to *ex vivo* generated Treg's after administration. Stability of the suppressive activity *in vivo*, post-administration is uncertain, given recent data that indicate unstable state *in vivo* (74, 78). Phenotypes in the *ex vivo*-generated Tregs that eventually-accumulate inside the disease target organ-draining lymph nodes, are also unclear. Are these Tregs directly involved in suppression of autoreactive T-effectors, or are they part of a network that responds to their presence whose comprehensive outcome is necessary to achieve some regulation of the underlying autoimmunity? Furthermore, *ex vivo*-generated Tregs, once administered into patients, begin to fall in numbers; circulating Treg levels fell to 25% maximal numbers in treated patients at 90 days in some studies (74). While some emerging planned trials are considering supplementing the Treg treatment with co-administration of IL-2 (NCT02772679), the level of CD25 on these cells (79) may limit the effect of the cytokine and instead further add to the survival and/or the stability of the Tregs once *in vivo*.

Two clinical trials have used *ex vivo*-expanded Treg cells for the treatment of new-onset disease, < 2 months, in T1D patients (NCT01210664, ISRCTN06128462) (74–77). These studies relied on Tregs generated from patient CD4+ CD25+ CD127- cells isolated from peripheral blood by flow cytometry and followed patients for 24 months post administration (74–77). Both studies examined patient C-Peptide levels as a marker for maintained insulin production, hence preserved beta-cell mass in the pancreas, as C-Peptide is cleaved from the proinsulin when it is converted to it's active insulin form. The first study maintained detectable C-peptide over the 2 year monitoring period, but revealed that circulating Treg levels fell to 25% at a peak of 90 days after infusion (74). During the same 90 day time frame the cell surface marker CD38, which has been associated with enhanced Treg function (80), dropped from >95% pre-infusion to < 5% post infusion. An additional phase I study is being planned to combine Treg administration with low-dose IL-2 treatment to see if a greater number of Treg can be maintained in T1D (NCT02772679). The second study displayed a transient increase in C-peptide with a reciprocal decrease in patient insulin usage. However, C-peptide values resumed a decline over the trial time-course (75–77). Plasma IL-6, a pro-inflammatory cytokine, was discovered to increase over the same 24 months in Treg treated patients, to levels detected in the untreated patients. A commercially-generated Treg population is also currently being tested in T1D (CLBS03; NCT02691247), however, at the time of our review, there were no results our outcomes publicly-disclosed. There are other reports indicating that autologous Treg therapy is in preparation for clinical trials in other conditions including autoimmune hepatitis (NCT02704338) and lupus (NCT02428309).

## Two is Better Than one: Combination Cell Therapy

While the possibility of combining tDC with Tregs as a co-administered or serially-administered cell therapy in autoimmunity, especially in new-onset T1D, would make scientific and therapeutic sense, thus far few if any have considered this. The inter-relationship of these cell populations on each other for functional outcomes, maintenance, stability, and “feed-forwarding” of a very powerful tolerogenic state should be self-evident. The autologous tDC, co-administered with the patient's Tregs, would stabilize Foxp3 expression as well as its genomic locus from the standpoint of the epigenome and, as tDC have often been shown to produce IL-10, TGFβ, and retinoic acid (66, 81, 82), the stabilized Tregs would in turn impact the tolerogenic state of the tDC via cell-cell interactions and paracrine immunoregulatory cytokines. In a potential treatment approach, the initial co-administration could be followed by periodic “boosters” of tDC and Tregs alone in serial administrations or be co-administered. While this makes mechanistic sense, the logistics to generate the cells would not necessarily be more challenging than they are now for the generation *ex vivo* of each product. For example, the leukapheresis that is part of the tDC generation protocols would cover the enrichment of monocytes, to generate the tDC as well as the initial step to collect the lymphocytes from which the Tregs would be expanded separately, in the same cell processing facility. As animal models of tDC and Treg cell therapy for autoimmunity including T1D are well-established, this possible co-therapy, we believe, is developed enough to investigate pre-clinically.

Considering the limitations and adverse events encountered using biologic agents and the need to move past systemically-acting immunosuppressives, the well-tolerated safety profile of tDC and Tregs, across a range of dose levels and administration sites, along with the evidence of increased regulatory cell frequency *in vivo* during treatment, strongly argues in favor of their further development, characterization and consideration, to fundamentally change how autoimmune diseases are treated, directly addressing the immune imbalance and moving away from disease and symptom management.

## Author Contributions

BP and NG wrote the manuscript. CE, MT, and YG edited the manuscripts and added additional insights. The final version was proofread and edited by NG.

### Conflict of Interest Statement

NG and MT hold equity in Diavacs Inc., which has licensed the intellectual property concerning the tolerogenic dendritic cells noted in the review under clinical trial numbers NCT00445913 and NCT02354911. The remaining authors declare that the research was conducted in the absence of any commercial or financial relationships that could be construed as a potential conflict of interest.

## Abbreviations

APC: Antigen-Presenting Cells
ATG: Anti-Thymocyte Globulin
Breg: B-regulatory cells
CD: Cluster of Differentiation
CTLA-4: Cytotoxic T-lymphocyte-Associated Protein 4
DC: Dendritic Cells
DC-10: High IL-10 secreting Dendritic Cells
Foxp3: Forkhead box protein P3
G-CSF: Granulocyte Colony Stimulating Factor
GM-CSF: Granulocyte Macrophage Colony Stimulating Factor
HbA1c: Hemoglobin A1c
HLA-DR: Human Leukocyte Antigen-antigen D Related
IDO, Indoleamine 2: 3-Dioxygenase
IFNγ: Interferon Gamma
IL: Interleukin
MHC: Major Histocompatibility Complex
MITAP: Minimum Information about Tolerogenic Antigen-Presenting cells
MPLA: Monophosphoryl Lipid A
NF-κB: Nuclear Factor kappa-light-chain-enhancer of activated B-cells
PBMC: Perpherial Blood Mononuclear Cells
PRR: Pattern Recognition Receptors
T1D: Type 1 Diabetes
TCR: T-Cell Receptor
tDC: Tolerogenic Dendritic Cells
Teff: T-Effector Cell
TGFβ: Transforming Growth Factor beta
TH, T-helper cells (1, 2: or 17)
TNFα: Tumor Necrosis Factor alpha
Tr1: IL-10 producing T-Regulatory cells
Treg: Foxp3+ T-Regulatory cells.
